# Adolescents suppress emotional expression more with peers compared to parents and less when they feel close to others

**DOI:** 10.1177/01650254221132777

**Published:** 2022-11-16

**Authors:** Megan S. Wylie, Kalee De France, Tom Hollenstein

**Affiliations:** 1 Queen’s University, Canada; 2 Yale University, USA

**Keywords:** Expressive suppression, social context, closeness with others, experience-sampling method

## Abstract

Adolescence is characterized by frequent emotional challenges, intense emotions, and higher levels of expressive suppression use than found in older populations. While evidence suggests that contingent expressive suppression use based on context is the most functional, it remains unclear whether adolescents use expressive suppression differentially based on social context. Because the peer relationship is highly salient in adolescence, the current study was designed to assess whether adolescents use expressive suppression differentially based on their social context. Adolescents (*N* = 179, *M_age_* = 13.94, 49.2% female) reported emotional events using experience sampling via a smartphone application for 14 days. Multilevel modeling revealed that adolescents used less expressive suppression when they were alone compared with when they were with people, and used more expressive suppression when they were with their peers compared with when they were with family. In addition, more closeness with family predicted less overall expressive suppression use, while closeness with peers did not influence the level of expressive suppression use within the peer context. We discuss the importance of peer relations in adolescence and the relationship between closeness and emotional expression.

## With Whom Do Adolescents Suppress Emotional Expression?

Adolescents experience frequent and intense emotions (Gross et al., 1997; Zarrett & Eccles, 2006) at a time when peer relations are rising in importance (Brown & Larson, 2009). Thus, maturing emotion regulation skills are critical and one of the skills they must improve is their emotional expression control, which is achieved mostly by using expressive suppression^1^ (Zimmerman & Iwanski, 2014, 2018). Suppression is a form of emotion regulation that involves the inhibition or concealment of emotional expressions (Gross et al., 2006). Suppression can be applied to a wide range of emotions, but a vast majority of the research using adult samples has specifically looked at the suppression of negative emotions (e.g., Butler et al., 2003; Gross, 1998). Being able to effectively suppress negative emotions is the hallmark of a well-regulated child (e.g., being able to control temper tantrums). Indeed, suppression continues to be an important skill in adolescence. For example, an adolescent may want to suppress expressions of anger while dealing with a frustrating, high-status peer. Furthermore, implementing suppression in a manner that is sensitive to context may be beneficial for psychosocial functioning (Bonanno et al., 2004; Greenaway et al., 2018; Gross & Cassidy, 2019; Kashdan & Rottenberg, 2010). Despite these important functional adaptions relevant to cultural display rules (Saarni, 1979), suppression is often cited as a maladaptive regulatory strategy due to its associations with poor psychosocial functioning when it is used excessively (i.e., without regard for context; (Aldao & Nolen-Hoeksema, 2010, 2012; John & Gross, 2004). However, it is unclear whether adolescents make important distinctions in their suppression use, and specifically whether they consider social context when using suppression. The purpose of the present study is to compare the degree to which adolescents discriminate between contexts in which they are alone, with parents, or with peers for the deployment of suppression for negative emotions in their day-to-day lives.

### Suppression Use in Adolescence

Although even young children know how to suppress their expressions (Gross & Cassidy, 2019), there is evidence that suppression use increases in adolescence (De France & Hollenstein, 2019; Zimmerman & Iwanski, 2014, 2018) for several possible reasons. First, adolescence is a developmental stage characterized by emotional challenges (Zarrett & Eccles, 2006), interpersonal conflicts (Laursen et al., 1998), and high intensity emotions (Gross et al., 1997), and expressive suppression is used most often to regulate high intensity emotions (Lennarz et al., 2019). Second, suppression requires relatively less effort to implement for high (and low) intensity emotions (i.e., compared with cognitive reappraisal; Sheppes & Gross, 2011), making it an easy strategy for adolescents to implement, while their emotion regulation skills are still developing. Third, heightened sensitivity to social rejection (Beeri & Lev-Wiesel, 2012) and greater concerns about the consequences of expressions in interpersonal contexts (Kalokerinos et al., 2014; Tafrate et al., 2002) may drive motivations to conceal emotional experiences (Gullone et al., 2010; Zimmerman & Iwanksi, 2014, 2018). Thus, developmentally, suppression may be more frequent and important in adolescence.

Different contexts can demand different expressive choices (Bonanno et al., 2007; Kennedy-Moore & Watson, 2001) and context-dependent and flexible suppression use is associated with positive psychosocial functioning (Bonanno et al., 2004; Gross & Cassidy, 2019; Kashdan & Rottenberg, 2010). Adults use suppression more with other people than when they are alone (English et al., 2017; Flores & Berenbaum, 2012; Jakobs et al., 2001), around strangers compared with people they know well (Benson et al., 2019; Chavez-Baldini et al., 2020), and children have been shown to express their emotions to differing degrees to parents and peers (Underwood et al., 1992; Zeman & Garber, 1996). However, although adolescent suppression has been shown to be contingent on at least one aspect of context, emotion intensity (Lennarz et al., 2019), it is not yet clear whether adolescents suppress expression based on who they are with at the time. Because of the shift from family-oriented to peer-oriented socioemotional contexts (Brown & Larson, 2009; Larson & Richards, 1991), adolescents seek ways to manage peer impressions (Saarni, 1999) and, therefore, may be motivated to suppress outward displays of their emotional experiences to ward off chastisement by peers (Salisch, 2001). In contrast, adolescents may be less motivated to suppress around their family, as they expect more supportive reactions to their negative expressions (Fuchs & Thelen, 1988; Zeman & Garber, 1996; Zeman & Shipman, 1997).

### The Present Study

The purpose of the present study was to contribute to our understanding of adolescent context-dependent suppression use with negative emotions in their day-to-day lives in three important ways, by (1) using novel measurement techniques to study this question, (2) assessing individual differences, and (3) examining gender differences.

First, we utilized experience-sampling method (ESM). Previous studies of regulatory habits have relied on trait-based assessments of suppression or social context, and these measures fail to capture variations in emotion regulation and instead rely on assumptions (i.e., that general tendencies reflect how people actually regulate on a day-to-day basis). Thus, capturing suppression and social context using ESM allows the assessment of individual variations in daily life. Because adults (English et al., 2017; Flores & Berenbaum, 2012; Jakobs et al., 2001) and children (Zeman & Garber, 1996) use less suppression when they are alone, we predicted that adolescents will also use less suppression when they are alone compared with when they are with others (Hypothesis 1a). Similarly, adults (Benson et al., 2019; Chavez-Baldini et al., 2020) and children (Fuchs & Thelen, 1988; Underwood et al., 1992; Zeman & Garber, 1996) express their emotions differentially depending on the specific social group they are around. Therefore, we predicted that adolescents would use more suppression when they are with peers compared with when they are with others (Hypothesis 1b), and they would use less suppression when they are with parents compared with when they are with others (Hypothesis 1c). In addition, relationships with peers are more salient and subject to more impression management than relationships with parents in adolescence (Saarni, 1999); therefore, we predicted that adolescents would use more suppression when they are with peers compared with when they are with parents (Hypothesis 1d).

Second, we considered individual difference factors in suppression use that have not been previously studied. Specifically, we were interested in exploring how closeness with others might affect general and context-dependent suppression use. Adults use suppression less when they are around people they feel close to (Benson et al., 2019), and there is some association between closeness and suppression use in adolescent–mother dyads (Martini & Busseri, 2012; Silva et al., 2018). However, this association has not been assessed with parents more generally, or with friends. We predicted that adolescents with greater closeness with friends and/or parents would use less suppression overall (Hypothesis 2a) and that closeness with each relationship type (friends or parents) would moderate the contingent suppression use in that social group (Hypothesis 2b). That is, parent closeness would moderate the parent versus other effect on suppression and closeness with friends would moderate the peer versus other effect on suppression.

Third, we considered gender differences in context-dependent suppression use that are not well understood. Males use suppression more than females (Flynn et al., 2010; Gross & John, 2003), as parents often encourage their sons to suppress negative emotions (Cassano et al., 2007). However, we do not know whether males and females differ in their context-dependent use of suppression. Therefore, we predicted that suppression use would differ by gender (Hypothesis 3).

## Method

### Participants

Participants for this study included 179 typically developing adolescents (49.2% female, 45.8% male), 12–15 years old (*M*_age_ = 13.94, *SD* = 0.88). Participants were recruited from a university participant database maintained by child development researchers located in southern Ontario, Canada. This database contains families from the community that volunteered to be included and consented to be contacted for developmental research studies. To be recruited for the current study, adolescents had to be 12–15 years old, and they had to have access to a mobile device (e.g., smartphone). Parents of participants provided consent and participants provided assent prior to participating in the study. Ethnic background was reported as follows: White (86.2%), Asian (13.7%), Black (2.3%), Latin American (2.9%), and other (2.3%). Ethnic background was representative of the city in which the study took place. Participants received $10 as compensation for completing the questionnaire portion of the study, plus participants received an additional $1 for every prompt they completed during the experience-sampling portion of the study, for a total possible compensation of $52. This study was approved by the Queen’s University General Research Ethics Board (GPSYC-817-17).

### Procedures

Participants and a parent attended laboratory sessions on a university campus. Participants then completed a series of online questionnaires, containing questions about friend and parent closeness. Then, a trained research assistant helped participants download the experience-sampling smartphone application (MetricWire; Trafford, 2015) that would prompt them to complete a short survey in the application three times per day for 2 weeks starting the following morning. Participants received prompts from the smartphone application at 11:00 am, 4:00 pm, and 8:30 pm for 14 consecutive days for a total of 42 prompts. We chose to use interval-contingent sampling as it allowed us to schedule prompts around participants’ standard daily activities (e.g., class time and after-school activities). Upon receiving the prompt, participants completed a 2- to 3-min survey. Participants were able to complete prompts within 90 min of receiving their notification, just in case they were unable to access their device when they first received the notification.^2^ After 90 min, the survey became inactive. Participants were not able to complete inactive surveys. Once the 14 days of experience sampling were complete, participants received an email containing a debriefing letter and were sent their study compensation via an electronic transfer.

### Measures

#### Friend and Parent Closeness

The Inventory of Parent and Peer Attachment (IPPA; Armsden & Greenberg, 1987) is a 53-item self-report questionnaire that captures relationship strength with parents and friends. Participants rated specific elements of their relationships, such as “I trust my friends” and “my parents respect my feelings,” on a 5-point scale from (1) almost never or never true to (5) almost always or always true. Friend Closeness was the mean across 25 friend relationship items; higher scores indicated higher friend attachment. Parent Closeness was the mean across 28 parent relationship items; higher scores indicate higher parent attachment. The IPPA friend (α = .95) and parent (α = .95) subscales had good reliability.

#### Suppression

For each mobile prompt, participants first reported which negative emotion^3^ they most recently experienced. Then, participants reported whether they used suppression in response to their most recent experience of a negative emotion. Participants answered the following question “What did you do in response to your emotion” and responded to one suppression item, “pretended I was not upset.” Responses were coded as (0) *Participant did not use suppression* or (1) *Participant did use suppression*.

#### Social Context

Participants reported who they were with during their most recent experience of a negative emotion. Participants were able to select any number of options from the following list: parents, siblings, teacher, students at school, staff at school, friends, boyfriend/girlfriend, boss, coworkers, other, I was alone.

### Variable Coding

Dummy coding was used to create four variables for Social Context: Alone, Peers, Family, and Peers versus Family. For the Alone variable, instances in which participants selected “alone” were coded as 1, and instances in which participants selected any other social context were coded as 0. Thus, this variable will compare being alone (1) versus not being alone (0). For the Peers variable, instances in which participants selected “friends,” “students at school” and/or “boyfriend/girlfriend” were coded as 1, and instances in which participants selected “siblings,” “teacher,” “staff at school,” “boss,” “coworkers,” and/or “other” were coded as 0. If participants selected “parents,” this variable was coded as missing. Thus, this variable will compare being with peers (1) versus being with others, not including parents (0). For the Parents variable, instances in which participants selected “parents” were coded as 1, and instances in which participants selected “siblings,” “teacher,” “staff at school,” “boss,” “coworkers,” and/or “other” were coded as 0. If participants selected “friends,” “students at school,” and/or “boyfriend/girlfriend,” this variable was coded as missing. Thus, this variable will compare being with parents (1) versus being with others, not including peers (0). For the Peers versus Parents variable, instances in which participants selected “friends,” “students at school,” and/or “boyfriend/girlfriend” were coded as 1, and instances in which participants selected “parents” were coded as 0. If participants selected “siblings,” “teacher,” “staff at school,” “boss,” “coworkers,” and/or “other,” this variable was coded as missing. Thus, this variable will compare being with peers (1) versus being with parents (0).

### Data Analysis Plan

We ran four different multilevel logistic models to predict suppression use for three social contexts: alone, with peers, and with parents. Models were run in M*plus* 8 using a multilevel framework due to the nested data^4^ (Muthén & Muthén, 2017). To build our models, we followed the suggestions outlined by Sommet and Morselli (2017). Friend and Parent Closeness were grand-mean centered (Raudenbush & Bryk, 2002). At Level 1 in each model, social context (Alone, Peers, Parents, and Peers vs Parents) predicted suppression use (Hypothesis 1a–d). At Level 2 in Models 2a, 2b, and 3, closeness with others (Friend and/or Parent) predicted suppression use (Hypothesis 2a) and the Level 1 slope of social context on suppression use (Hypothesis 2b). Finally, gender comparisons will be run in each model (Hypothesis 3).

In Model 1, we assessed the effect of being alone compared with not being alone. At Level 1, Suppression was predicted by Alone (0/1) (Hypothesis 1a). Model equations were as follows:

*Level 1*

$$\mathrm{Suppression}\leftarrow b_{0}+b_{1}\left(\mathrm{Alone}\right)+\mathrm{error}$$

In Model 2a, we assessed the effect of being with peers compared with being with others (not including parents). At Level 1, Suppression was predicted by Peers (0/1) (Hypothesis 1b), and at Level 2 Friend Closeness predicted Suppression (Hypothesis 2a), and the slope of Peers on Suppression (Hypothesis 2b). Model equations were as follows:

*Level 1*

$$\mathrm{Suppression}\leftarrow b_{0}+b_{1}\left(\mathrm{Peers}\right)+\mathrm{error}$$

*Level 2*

$$\begin{array}{l} b_{0}\leftarrow \gamma_{00}+\;\gamma_{01}\mathrm{Friend}\;\mathrm{Closeness}\;+\;\mathrm{error}\\ b_{1}\leftarrow \gamma_{10}+\gamma_{\mathrm{11}}\mathrm{Friend}\mathrm{Closeness}+\mathrm{error} \end{array}$$

In Model 2b, we assessed the effect of being with parents compared with being with others (not including peers). At Level 1, Suppression was predicted by Parents (0/1) (Hypothesis 1c), and at Level 2 Parent Closeness predicted Suppression (Hypothesis 2a) and the slope of Parents on Suppression (Hypothesis 2b). Model equations are the same as above, replacing any Peer variables with Parent variables.

In Model 3, we assessed the effect of being with peers compared with being with parents. At Level 1, Suppression was predicted by Peers versus Parents (Hypothesis 1d), and at Level 2 Friend and Parent Closeness predicted Suppression (Hypothesis 2a), and the slope of Peers versus Parents on Suppression (Hypothesis 2b). Model equations are as follows:

*Level 1*

$$\mathrm{Suppression}\leftarrow b_{0}+b_{1}\left(\mathrm{Peers}\mathrm{vs}.\mathrm{Parents}\right)+\mathrm{error}$$

*Level 2*

$$\begin{array}{l} b_{0}\leftarrow \gamma_{00}+\gamma_{01}\mathrm{Friend}\mathrm{Closeness}+\mathrm{error}\\ b_{0}\leftarrow \gamma_{00}+\gamma_{02}\mathrm{Parent}\mathrm{Closeness}+\mathrm{error}\\ b_{1}\leftarrow \gamma_{10}+\gamma_{\mathrm{11}}\mathrm{Friend}\mathrm{Closeness}+\mathrm{error}\\ b_{1}\leftarrow \gamma_{10}+\gamma_{\mathrm{12}}\mathrm{Parent}\mathrm{Closeness}+\mathrm{error} \end{array}$$

Finally, we also assessed gender differences in suppression use by running all four models separated by gender,^5^ Models 1 to 3 were each run for males only and for females only (Hypothesis 3).

## Results

Preliminary analyses were run in SPSS (IBM Corp., 2019) to obtain descriptive values and correlations. First, we calculated each individual’s mean suppression use across the 42 prompts, indicating what percent of prompts individuals used suppression. This variable ranged from 0 (suppression was never used) to .98 (suppression was used 98% of the time), and the mean of this variable was .27 (*SD* = 0.21), indicating that on average, participants used suppression at 27% of their prompts. Second, each participant’s proportion of Suppression prompts in which they were Alone, with Peers, and with Parents were calculated. We then calculated the means and standard deviations for these three variables, which could range from 0 (never use suppression in a given social context) to 1 (only use suppression in a given social context). The mean indicates the average proportion of an individual’s suppression prompts in which they were in a given social context. The average proportion of suppression prompts in which participants were alone was 11%, *M* = 0.11, *SD* = 0.22. The average proportion of suppression prompts in which participants were with peers was 33%, *M* = 0.33, *SD* = 0.33. The average proportion of suppression prompts in which participants were with parents was 37%, *M* = 0.37, *SD* = 0.32. Third, the correlation between Friend Closeness, *M* = 3.81, *SD* = 0.72, and Parent Closeness, *M* = 3.71, *SD* = 0.77, was positive, *r* = .39, *p* < .01.

Results for all multilevel models can be found in Table 1. In Model 1, consistent with Hypothesis 1a suppression use was less likely when participants reported being alone. The odds ratio indicated that when adolescents were alone they were 29% less likely to use suppression than when they were with other people.

**Table 1.** Multilevel Model Results.

Model	Level	Effect	*b*(*SE*)	*p*	ULCI/LLCI	Odds ratio
Model 1	Level 1	**Alone → Suppression (*b*_1_)**	**−0.34(0.14)**	**.02**	**−0.62, −0.06**	**0.71**
Model 2a	Level 1	Peer → Suppression (*b*_1_)	0.22(0.17)	.20	−0.12, 0.55	1.24
	Level 2	Friend Closeness → Suppression	−0.38(0.20)	.06	−0.77, 0.01	0.68
		Friend Closeness → *b*_1_	−0.03(0.23)	.90	−0.47, 0.41	0.97
Model 2b	Level 1	Parent → Suppression (*b*_1_)	−0.28(0.15)	.05	−0.57, 0.002	0.75
	Level 2	**Parent Closeness → Suppression**	**−0.57(0.18)**	**.001**	**−0.92, −0.22**	**0.57**
		Parent Closeness → *b*_1_	−0.06(0.17)	.72	−0.40, 0.28	0.94
Model 3	Level 1	**Peer vs Parent → Suppression (*b*_1_)**	**0.36(0.12)**	**.003**	**0.12, 0.60**	**1.44**
	Level 2	Friend Closeness → Suppression	−0.19(0.15)	.21	−0.49, 0.11	0.82
		**Parent Closeness → Suppression**	**−0.50(0.15)**	**.001**	**−0.80, −0.21**	**0.60**
		Friend Closeness → *b*_1_	0.03(0.14)	.84	−0.25, 0.30	1.03
		Parent Closeness → *b*_1_	0.14(0.16)	.40	−0.18, 0.45	1.15

*Note. N* = 179; *SE*: standard error; ULCI: upper-level 95% confidence intervals; LLCI: lower-level 95% confidence intervals. Betas are unstandardized. Bolded lines are significant effects.

In Model 2a, not consistent with Hypothesis 1b, being with peers did not significantly predict suppression use. In addition, Friend Closeness did not predict suppression use, inconsistent with Hypothesis 2a. Finally, not consistent with Hypothesis 2b, the cross-level interaction between Friend Closeness and Peers was not statistically significant.

In Model 2b, not consistent with Hypothesis 1c, being with parents did not significantly predict suppression use. However, consistent with Hypothesis 2a, Parent Closeness negatively predicted suppression use, and the odds ratio indicated that for every 1 unit increase in Parent Closeness, adolescents were 43% less likely to use suppression regardless of who they were with at the time. Finally, not consistent with Hypothesis 2b, the cross-level interaction between Parent Closeness and Parents was not statistically significant.

In Model 3, consistent with Hypothesis 1d, being with peers significantly predicted suppression use, and the odds ratio indicated that when adolescents were with peers, they were 1.4 times more likely to use suppression, compared with when they were with parents. Friend Closeness did not predict suppression use, but Parent Closeness did significantly predict suppression use, partially consistent with Hypothesis 2a. The odds ratio indicated that for every 1 unit increase in Parent Closeness, adolescents were 40% less likely to use suppression regardless of who they were with at the time. The cross-level interaction between Friend Closeness and Parent Closeness and Peers versus Parents was not statistically significant, not consistent with Hypothesis 2b.

Finally, we ran each of our models for male and female participants independently to determine whether there were any gender differences in suppression use based on social context and closeness with others (results can be found in Tables 2 and 3). In Model 1, being alone predicted less suppression use for males only. In Model 2a, Friend Closeness predicted less suppression use regardless of who they were with at the time for males only. In Model 2b, Parent Closeness predicted less suppression use regardless of who they were with at the time for both females and males. In Model 3, being with peers predicted more suppression use for females only, and Parent Closeness predicted less suppression use regardless of who they were with at the time for both females and males. Results were consistent with Hypothesis 3.

**Table 2.** Multilevel Model Results: Females.

Model	Level	Effect	*b*(*SE*)	*p*	ULCI/LLCI	Odds ratio
Model 1	Level 1	Alone → Suppression (*b*_1_)	−0.19(0.20)	.35	−0.58, 0.21	0.83
Model 2a	Level 1	Peer → Suppression (*b*_1_)	0.30(0.21)	.15	−0.11, 0.71	1.35
	Level 2	Friend Closeness → Suppression	0.15(0.22)	.51	−0.29, 0.58	1.16
		Friend Closeness → *b*_1_	−0.30(0.30)	.32	−0.90, 0.29	0.74
Model 2b	Level 1	Parent → Suppression (*b*_1_)	−0.30(0.17)	.07	−0.63, 0.02	0.74
	Level 2	**Parent Closeness → Suppression**	**−0.35(0.18)**	**.05**	**−0.70, −0.002**	**0.70**
		Parent Closeness → *b*_1_	−0.01(0.17)	.93	−0.34, 0.32	0.99
Model 3	Level 1	**Peer vs Parent → Suppression (*b*_1_)**	**0.52(0.16)**	**.001**	**0.21, 0.82**	**1.68**
	Level 2	Friend Closeness → Suppression	0.10(0.23)	.66	−0.35, 0.55	1.11
		**Parent Closeness → Suppression**	**−0.37(0.17)**	**.03**	**−0.71, −0.03**	**0.69**
		Friend Closeness → *b*_1_	−0.19(0.19)	.31	−0.56, 0.18	0.83
		Parent Closeness → *b*_1_	0.34(0.19)	.07	−0.03, 0.71	1.41

*Note. N* = 88; *SE*: standard error; ULCI: upper-level 95% confidence intervals; LLCI: lower-level 95% confidence intervals. Betas are unstandardized. Bolded lines are significant effects.

**Table 3.** Multilevel Model Results: Males.

Model	Level	Effect	*b*(*SE*)	*p*	ULCI/LLCI	Odds ratio
Model 1	Level 1	**Alone → Suppression (*b*_1_)**	**−0.53(0.21)**	**.01**	**−0.93, −0.13**	**0.59**
Model 2a	Level 1	Peer → Suppression (*b*_1_)	0.07(0.27)	.80	−0.46, 0.59	1.07
	Level 2	**Friend Closeness → Suppression**	**−0.97(0.28)**	**.00**	**−1.51, −0.43**	**0.38**
		Friend Closeness → *b*_1_	0.08(0.27)	.78	−0.45, 0.61	1.08
Model 2b	Level 1	Parent → Suppression (*b*_1_)	−0.26(0.26)	.32	−0.76, 0.25	0.78
	Level 2	**Parent Closeness → Suppression**	**−0.95(0.32)**	**.003**	**−1.57, −0.33**	**0.39**
		Parent Closeness → *b*_1_	−0.20(0.40)	.61	−0.98, 0.57	0.82
Model 3	Level 1	Peer vs Parent → Suppression (*b*_1_)	0.11(0.19)	.56	−0.26, 0.48	1.12
	Level 2	Friend Closeness → Suppression	−0.46(0.25)	.07	−0.96, 0.04	0.63
		**Parent Closeness → Suppression**	**−0.71(0.31)**	**.02**	**−1.31, −0.11**	**0.49**
		Friend Closeness → *b*_1_	0.17(0.25)	.49	−0.32, 0.67	1.19
		Parent Closeness → *b*_1_	−0.22(0.28)	.43	−0.78, 0.33	0.80

*Note. N* = 82; *SE*: standard error; ULCI: upper-level 95% confidence intervals; LLCI: lower-level 95% confidence intervals. Betas are unstandardized. Bolded lines are significant effects.

## Discussion

The objective of the current study was to understand momentary suppression use in relation to social context and closeness with others. We used an experience-sampling design and found that adolescents did use suppression based on who they are with, and better closeness with parents predicted less suppression use consistently, while better closeness with friends predicted less suppression use for males only.

### Social Context

As predicted, adolescents used less suppression when they were alone compared with when they were with other people. There are no interpersonal consequences associated with expressing negative emotions when you are alone (Kalokerinos et al., 2014; Tafrate et al., 2002), and it seems that adolescents understand that they do not need to hide their emotional expressions in this context. Adolescents did not use more or less suppression when they were with peers or parents compared generally to other people, contrary to predictions. However, adolescents did use more suppression when they were with peers compared to when they were with parents, as predicted. Peer relationships are more salient than parent relationships in adolescence, and adolescents are highly motivated to manage peer impressions (Saarni, 1999). Given the possible negative interpersonal consequences associated with expressing negative emotions (Kalokerinos et al., 2014; Tafrate et al., 2002), adolescents may deploy suppression to avoid these consequences when they are with their peers. These results indicate that adolescents choose whom to express their emotions around, and they seem to do this particularly with their peers.

### Closeness with Others

As predicted, closeness with parents, but not peers, negatively predicted suppression use; adolescents generally express their emotions more when they feel close with their parents and less when they do not feel close with their parents. There are two possibilities as to which end of the distribution may be driving this effect. On one hand, feeling close with others implies greater trust and less uncertainty about how others feel (Salazar, 2015). This may motivate adolescents to express their emotions across many social contexts, as they may feel comfortable sharing their genuine emotional experiences. Previous studies suggest similar patterns, as secure attachment with others is associated with less suppression use in adolescence and adulthood (Karreman & Vingerhoets, 2012). On the other hand, not feeling close with others implies less trust and more uncertainty about how others feel (Salazar, 2015). Lower closeness may motivate adolescent concerns or uncertainties about how others in or outside the family will respond to their emotional expressions, such as fear of rejection (London et al., 2007; Marston et al., 2010). Moreover, greater suppression use may further limit adolescents’ ability to enhance closeness with others (Tackman & Srivastava, 2016). In either case, because parents provide the foundational emotional climate for children and adolescents to learn about and explore their emotional expressions (Halberstadt et al., 1999; Root & Denham, 2010), the current results indicate this developmental experience may extend to suppression use.

Contrary to our hypothesis, closeness with friends did not predict suppression use; feeling close with friends did not impact how much adolescents express or suppress their emotions. These results suggest that, despite the importance of peer relationships in adolescence (Brown & Larson, 2009), feeling close with friends does not influence adolescents’ emotional expressions. These results could, in part, be due to conceptual differences between closeness and social context, as we looked at closeness with friends specifically and the peer context more generally. Adolescents may differentiate their attachment with different peer groups, so there may have been a more specific peer effect that we did not capture in this study. It would be valuable for future studies to consider specific peer contexts more closely, as well as considering situation (e.g., being in the classroom versus at the mall).

Finally, closeness with others did not moderate contingent suppression use as we had expected: feeling close with peers did not predict how much adolescents would suppress around their peers and feeling close with parents did not predict how much adolescents would suppress around their parents. Perhaps this is a distinction that emerges later, as adolescents may lack the ability to consider how close they feel with someone while they are attempting to regulate their emotions around that person. Alternatively, as only parent closeness predicted overall suppression use, it may be that this accounted for so much of the variance that there was no moderation effect.

### Gender Differences

We found that only males used less suppression when they were alone compared with when they were with other people, and only females used more suppression when they were with their peers compared with when they were with their parents. In addition, closeness with friends influenced suppression use for males and not for females. Taken together, these results indicate that females did not use less suppression when they were alone, but they did specifically increase their suppression use around peers and did so regardless of how close they generally felt with peers. Female adolescents may use suppression more around their peers to protect themselves (Salisch, 2001) from negative psychosocial problems (i.e., greater depression and lower self-esteem) associated with peer relationships that male adolescents are less sensitive to (Moran & Eckenrode, 1991).

### Limitations

This study does present some limitations. As with any experience-sampling study, we had to make some design choices that limited the amount of information we could capture at a given prompt. Specifically, we only used one binary item per prompt to capture expressive suppression use (suppression not used or suppression used). Individuals may have different thresholds for suppression use, so at a given prompt, what might be considered suppression use for one person may not be considered suppression use for another. Recent studies have tested multiple Likert-type scale items to capture emotion regulation strategy use and have found within and between person reliability (Medland et al., 2020). In addition, we only captured one aspect of suppression: frequency. Perhaps other aspects, such as effort or motivation, of one’s suppression attempt may be relevant for social context and closeness with others. This topic warrants future research. Finally, due to limited power and limitations within MPlus, we were unable to formally test the moderation effect of gender, which may have produced slightly different gender results.

## Conclusion

We have demonstrated that adolescents engage in suppression based on social context and how close they feel with others. This study warrants replication and expansion, specifically, analyzing specific social groups independently (e.g., parents vs siblings, classmates vs close friends) to better understand the nuance of this distinction in adolescence. Likewise, research making comparisons from childhood to adulthood could establish when context-dependent suppression use is developed. The current study represents an important first step in understanding social influences on expressive habits in adolescence.

## Notes

1. From this point on, we will refer to expressive suppression simply as suppression.

2. Adolescents do not all have the same schedule (e.g., some schools stagger lunch breaks), so we opted for a large window of time to accommodate these differences in daily routines while still administering surveys relatively close together (i.e., a mid-day survey, an afternoon survey, and an evening survey).

3. We chose to make negative emotions the focus of this study as a majority of the research on emotion regulation focuses on the regulation of negative emotions. However, we do not want to suggest that people do not suppress their positive emotions, as there are many reasons one might want to conceal positive expressions.

4. Additional study details including compliance and data cleaning can be found at the following OSF link: https://osf.io/sd6zn/

5. We chose to run two models separated by gender as we were unable to run a group comparison in MPlus using type = mixture model and estimator = MLR and we were concerned about power for effect comparison tests (e.g., Wald’s tests).
